# Cost-effectiveness of hypertension treatment: a population-based study

**DOI:** 10.1590/S1516-31802002000400002

**Published:** 2002-07-07

**Authors:** Juvenal Soares Dias da Costa, Sandra Costa Fuchs, Maria Teresa Anselmo Olinto, Denise Petrucci Gigante, Ana Maria Baptista Menezes, Silvia Macedo, Sabrina Gehrke

**Keywords:** Cost-effectiveness, Hypertension, Treatment, Diuretics, Beta blockers, Custoefetividade, Hipertensão, Arterial, Tratamento, Diuréticos, Betabloqueadores

## Abstract

**CONTEXT:**

The cost-effectiveness of the treatment of hypertension has scarcely been investigated in population-based studies. Most data come from secondary analysis of clinical trials and administrative sources.

**OBJECTIVE:**

To describe the healthcare costs for outpatient hypertension treatment in comparison with diabetes mellitus and chronic bronchitis, and to examine the cost-effectiveness of different classes of antihypertensive drugs.

**DESIGN:**

Cross-sectional population-based study.

**SETTING:**

Urban area of Pelotas, southern Brazil.

**PARTICIPANTS:**

Individuals aged 20-69 years, identified through multi-stage probability sampling.

**METHODS:**

Participants were interviewed at home. Demographic data, education, income, smoking, previous morbidity, use of medicine and other characteristics were assessed via a pre-tested questionnaire, and blood pressure while seated was measured in a standardized way.

**RESULTS:**

Approximately 24% of the participants had high blood pressure or were taking antihypertensive drugs, and among these, 33% had had a physician consultation during the month preceding the interview. The monthly mean costs of care for hypertension (R$ 89.90), diabetes (R$ 80.64) and bronchitis (R$ 92.63) were similar. Treatment of hypertension consumed 22.9% of the per-capita income, corresponding to R$ 392.76 spent per year exclusively on antihypertensive drugs. Most of the direct costs associated with hypertension and diabetes were spent on drugs, while patients with bronchitis had greater expenditure on appointments. The cost-effectiveness relationship was more favorable for diuretics (116.3) and beta blockers (228.5) than for ACE inhibitors (608.5) or calcium channel blockers (762.0).

**CONCLUSION:**

The costs of hypertension care are mainly dependent on the expenditure on blood pressurelowering drugs. Treatment of hypertension with diuretics or beta blockers was more cost-effective than treatment with ACE inhibitors and calcium channel blockers.

## RESUMO

**CONTEXTO:**

O custo-efetividade do tratamento da hipertensão tem sido pouco investigado em estudos de base populacional. A maior parte do conhecimento nesta área provém de dados sobre participantes de ensaios clínicos randomizados e do controle administrativo.

**OBJETIVO:**

Descrever os custos com a atenção à saúde para o tratamento da hipertensão em comparação com diabetes mellitus e bron- quite crônica e examinar o custo-efetividade de diferentes classes de anti-hipertensivos, desconsiderando admissão hospitalar.

**TIPO DE ESTUDO:**

estudo transversal de base populacional.

**LOCAL:**

Região urbana de Pelotas, sul do Brasil.

**PARTICIPANTES:**

Indivíduos com idade entre 20-69 anos, identificados por meio de uma amostra probabilística por estágios múltiplos.

**MÉTODOS:**

Participantes foram entrevistados no domicílio. Dados demográficos, sobre educação, renda, tabagismo, morbidade pré- via, uso de medicamentos e outras características foram avaliados por um questioná- rio pré-testado, sendo a pressão arterial aferida com o indivíduo sentado e de ma- neira padronizada.

**RESULTADOS:**

Aproximadamente 24% dos participantes tinham pressão alta ou faziam uso de anti-hipertensivos e, entre eles, 33% tinham realizado consulta médica no mês precedente à entrevista. O custo médio men- sal do cuidado com a hipertensão (R$ 89,90), diabetes (R$ 80,64) e bronquite (R$ 92,63) foi semelhante. O tratamento da hiperten- são consumiu 22,9% da renda per capita, correspondendo a R$ 392,76 gastos anualmente exclusivamente em medicamentos anti-hipertensivos. A maior parte dos custos diretos associados com hipertensão e diabetes foi devida a medicamentos, enquanto pacientes com bronquite crônica tiveram mais despesas com consultas. A relação de custo- efetividade foi mais favorável para diuréticos (116.3) e betabloqueadores (228.5) do que para inibidores da enzima de conversão da angiotensina (608.5) ou bloqueadores dos canais de cálcio (762.0).

**CONCLUSÃO:**

O custo do cuidado ambulatorial com a hipertensão foi dependente principalmente do tratamento anti-hipertensivo. O tratamento da hipertensão com diuréticos ou betabloqueadores foi mais custo-efetivo do que o tratamento com inibidores da enzima de conversão de angiotensina e bloqueadores dos canais de cálcio.

## INTRODUCTION

Hypertension is a prevalent chronic disease strongly related to the development of cerebrovascular and ischemic heart disease. Prevalence of hypertension (blood pressure^3^ 160/95 mmHg or antihypertensive drug use) ranged from 19.2 to 29.4% among Brazilian population-based surveys.^1-4^ It has been estimated that less than 20% of hypertensive patients have adequate control of blood pressure.^5^ Even though randomized clinical trials have determined the efficacy of antihypertensive treatment, the effective control of hypertension depends on case detection and adequate management by health professionals, followed by the long-term adhesion of patients to the treatment.^6^ Antihypertensive drug treatment often has elevated costs,^7^ a limitation that has not always been taken in account in clinical practice.^8^

In Brazil, most data on the costs of chronic disease treatment come from administrative sources, such as the number of hospital admissions, medical procedures and medical consultations. Cost-effectiveness analysis is seldom available, particularly with regard to the individualization of costs.^9^

In this report we describe the components of the healthcare cost for individuals with hypertension identified in a population-based survey, and the costeffectiveness relationship of antihypertensive treatment. In order to assess the economic burden of the treatment of hypertension, the costs were compared with those of diabetes and bronchitis treatment.

## METHODS

### Design

A cross-sectional population-based study was carried out in the metropolitan area of Pelotas (RS) from December 1999 to April 2000. The main objective was to investigate characteristics associated with healthcare and their costs in the adult population aged 20 to 69 years. The research protocol was approved by the Research and Ethics Committee of the Faculty of Medicine, Universidade Federal de Pelotas.

### Sampling and sample size

The participants were randomly selected through cluster sampling from 40 census sections, i.e. limited geographical zones of the city defined by the Brazilian Institute of Geography and Statistics (IBGE). In each section, a starting point of one street block was picked at random and the first house was identified, followed by systematic sampling of the next 30 houses. A total of 1,200 families with 1,800 individuals were expected to be identified.

A sample size of 1,800 individuals would be capable of detecting a prevalence ratio of 1.6, with 80% power and 5% alpha error, for presentations ranging between 25% and 75%. An additional 10% of participants was included to account for potential refusals, and a further 15% to ensure power for the multivariate analysis. In total, 1,257 families were identified, of whom 57 did not fulfill the age criterion. From the 1,200 eligible families, 1,145 (95.4%) were studied and 4.5% could not be reached or refused to participate. A total of 2,177 persons were identified and 1,968 interviewed (90.4%). In this analysis, all participants with hypertension (presenting blood pressure ≥ 160/95 mmHg or taking antihypertensive drugs), with diagnosis of bronchitis or diabetes were included. The costs of each item were based on the reports by participants who had had a medical appointment within the month preceding the interview. The costeffectiveness analysis included participants with hypertension under treatment with antihypertensive drugs.

### Interview Procedures

Participants were interviewed and blood pressure was measured at home, after informed consent was obtained. Interviewers were trained and certified in the techniques of interviewing and measurement of blood pressure.^10^ The blood pressure was measured using aneroid sphygmomanometers calibrated against a mercury tensiometer according to the Brazilian guidelines.^10^ A standardized pretested pre-coded questionnaire was used to collect data on demographics, socioeconomics, morbidity, healthcare and use of drugs.

The questionnaires were reviewed by the supervisors, who repeated 10% of the interviews at random using a short version of the questionnaire.

### Diagnosis of hypertension and other chronic diseases

Hypertension was characterized as blood pressure ≥ 160/95 mmHg (from an average of two measurements), or the use of antihypertensive drugs. This cutoff was adopted in order to reduce the potential for bias in the measurements through the phenomenon of regression to the mean. Individuals on antihypertensive drug treatment whose systolic blood pressure was lower than 160 mmHg and diastolic blood pressure was lower than 95 mmHg were considered as having controlled hypertension. Diabetes mellitus was identified based on an existing diagnosis.

Chronic bronchitis was characterized by cough with sputum during most days of the month, for at least three months, for two consecutive years.^11^

### Cost Analysis

Participants who had had a consultation during the month preceding the interview were asked about direct healthcare costs, including the purchase of drugs or supplies, payment for visits to doctors, laboratory tests, health insurance costs, and expenses with meals and transportation to the healthcare facility. Indirect costs were investigated through absenteeism (workdays lost) due to disease, medical consultations, or performing tests. The questionnaire also sought information on the trade name for each medicine, and its dose and interval. The costs of the antihypertensive drug therapies were calculated as a function of the dosage prescribed and the prices in the Pharmacy Guide Magazine (*Revista Guia da Farmácia*), April 2001. The overall cost of each class of antihypertensive was estimated as the mean cost of that class.

The expenditure on health insurance and laboratory tests was reported by the patient. The monthly cost of these items was considered as a direct expense, independent of whether it had been used during the preceding month. Expenses with meals, transportation and laboratory tests were also considered as direct expenses. Indirect costs due to productivity losses caused by partial or total absence from work were estimated via the proportional per-capita income earned during one working day. The total cost was the sum of the preceding items.

The costs of antihypertensive treatment were based on reports from 259 participants regarding monthly expenditure on drugs. The cost-effectiveness relationship of antihypertensive treatment was described on the basis of annual cost.

### Data analysis

Questionnaires were coded by the interviewers and checked by the research assistant for completeness before making a double data entry. Epi Info software was used to generate a database file, and the Statistical Package for the Social Sciences (Chicago, IL), version 8.0 for Windows, and Microsoft Excel software were used in the analysis.

Direct and indirect costs were described using means and standard deviations in order to compare the expenses of hypertension patients with those of chronic bronchitis and diabetes mellitus patients. The proportion of participants with blood pressure < 160/95 mmHg was calculated for each group of blood pressure-lowering drugs.

The cost-effectiveness relationship was calculated as a ratio of the annual mean cost to the proportion of patients with controlled hypertension, for each pharmacological group. The cost-effectiveness ratio allowed the cost per patient with controlled hypertension to be described.^12^ Since patients with two or three chronic conditions (hypertension, diabetes and smoking) were more prone to spend money on drugs, to have lower degrees of adhesion and to have uncontrolled hypertension, the cost-effectiveness analysis was additionally stratified by the presence of these comorbidities.

## RESULTS

Among the 1,968 participants interviewed, 462 (23.5%) had blood pressure ≥ 160/95 mmHg or were taking antihypertensive drugs. These individuals had a mean age of 52.5 ± 10.5 years, had 6.7 ± 4.6 years of school education and were predominantly female (73%). Among the 154 participants who had had a medical appointment during the month preceding the interview, 20% were unaware of their high blood pressure and 3.2% of patients with known hypertension were not taking antihypertensive drugs.

Table 1 shows the direct and indirect costs for treating hypertension, diabetes mellitus and chronic bronchitis. Most of the direct costs associated with hypertension and diabetes were due to expenditure on drugs, health insurance plans and medical appointments, while patients with bronchitis had greater expenditure on consultations and drugs. Laboratory tests represented a greater cost for patients with bronchitis than for those with diabetes or hypertension. The total cost of chronic bronchitis treatment was greater than for patients with diabetes or hypertension.

**Table 1:** Monthly mean costs to patients of healthcare components in the treatment of hypertension, diabetes mellitus and chronic bronchitis, in Pelotas, Brazil (2002), in Reais (R$)

	Hypertension (N=154)	Diabetes mellitus (N=46)	Chronic bronchitis (N=30)
**Direct costs**			
Drugs	32.73	28.46	21.80
Health insurance	23.35	20.52	6.57
Medical consultation	22.21	21.00	47.18
Laboratory tests	4.68	2.46	12.43
Transportation and meals	2.28	2.10	1.78
**Indirect cost**			
Loss of productivity	4.64	6.06	2.87
Mean (± SD)	89.90 (± 128.56)	80.64(± 245.22)	92.63(± 254.22)

Treatment of hypertension consumed 22.9% of the per-capita income, corresponding to R$ 392.76 spent per year exclusively on blood pressure-lowering drugs.

Table 2 presents the cost-effectiveness relationship of the antihypertensive treatment with medicines. Diuretics and beta blockers were the drugs most frequently used in monotherapy, while the most common associations were diuretics and beta blockers or diuretics and angiotensin-converting enzyme (ACE) inhibitors. The cost of antihypertensive treatment was lower for diuretics and beta blockers in monotherapy or associations, but only 55% of the patients taking diuretics had blood pressure < 160/ 95 mmHg. Overall, the cost-effectiveness relationship was more advantageous for diuretics and beta blockers than for the ACE inhibitors or calcium channel blockers. However, the cost-effectiveness analysis of antihypertensive treatment was markedly different among hypertensive patients with (n = 65) or without (n = 194) comorbidities (diabetes or smoking). Among patients with hypertension and diabetes or smoking, a less favorable relationship was detected for monotherapy with beta blockers (321.00 vs. 215.28) and diuretics (127.98 vs. 109.88) or in association (388.06 vs. 299.15). Patients without comorbidities presented a less advantageous ratio for ACE inhibitors (869.73 vs. 487.73) and calcium channel blockers (1052.59 vs. 629.68).

**Table 2:** Cost-effectiveness of antihypertensive treatment in Pelotas, RS, 2002

Antihypertensive treatment	N =259 (%)	Annual mean costs (R$)	% Patients with controlled hypertension (95% CI)	Cost-effectiveness Ratio
Diuretics	71 (27.4)	63.84	54.9 (43.3-66.5)	116.3
Beta blockers	31 (12.0)	162.24	71.0 (55.0-86.9)	228.5
Calcium channel blockers	10 (3.9)	609.60	80.0 (55.2-104.7)	762.0
ACE inhibitors	25 (9.7)	316.44	52.0 (32.4 -71.6)	608.5
Diuretics + Beta blockers	36 (13.9)	161.88	55.6 (39.3-71.8)	291.2
Diuretics + Calcium channel blockers	13 (5.0)	531.12	61.5 (35.1-88.0)	863.6
Diuretics + ACE Inhibitors	30 (11.6)	459.60	36.7 (19.4-53.9)	1252.3
Beta blockers + Calcium channel blockers	6 (2.3)	522.72	50.0 (10.0-90.0)	1045.4
Beta blockers + ACE Inhibitors	3 (1.2)	622.68	66.7 (13.3-120.0)	933.6
Other combinations	34 (13.1)	654.24	47.0 (30.3-63.8)	1392.0

**Figure 1:**
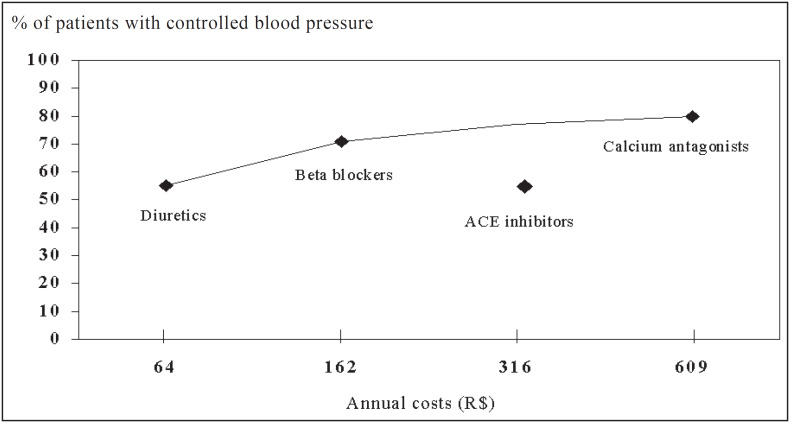
Cost-effectiveness relationships for anti-hypertensive monotherapies.

Figure 1 shows that the increase in annual costs accounts for a proportionally higher level of hypertension control for most antihypertensive drugs, but not for ACE inhibitors.

## DISCUSSION

This study was able to describe the cost of the treatment and control of hypertension for patients taking blood pressure-lowering drugs. The selection of a population-based sample has the advantage of including a representative sample of the entire population and allows the cost-effectiveness of treatment based on drugs actually in use to be assessed, thereby differing from indirect estimates based upon data from production and sales of drugs, medical records^12^ or participants in randomized clinical trials.^13^ Each component of the cost was determined using direct information from the individuals under medical care for hypertension, diabetes and bronchitis. The investigation of costs over a period of one month was employed to avoid recall bias, although information on a longer period might have taken into account procedures performed occasionally. Otherwise, the expenditure on drugs represents the average month expenses based on an index month.

The lack of information on hospital costs is a limitation of this study, since hospital admission expenses were not covered and these represent the greatest cost.^14,15^ Despite the fact that expenditure on the acquisition of antihypertensive drugs is a poor predictor of the total cost of treatment,^8^ there is no data regarding individual expenditure on hypertension treatment in Brazil.

The analysis of the components of total cost showed that the purchase of antihypertensive drugs accounted for 36% of the whole amount spent by hypertension patients. The low expenditure on medical appointments and laboratory tests may be attributed to the fact that most people use the public health system.^16^

In a previous study using this population, 30% of the participants were unaware of hypertension and, among those who had hypertension, 10% were not taking antihypertensive drugs.^17^ The presumed better control of hypertension, with 3.2% not taking antihypertensive drugs in the present study versus 10% in the earlier study, may be secondary to the sub-sampling criterion, since it is likely that participants who had previously had a medical appointment may have been more concerned with health than the general population. The requirement to have had a recent consultation probably explains the over-representation of women in the analysis of cost components, since they are more likely to seek healthcare than men.^18^ In addition, 20% of the individuals who had had a recent medical appointment were unaware of the diagnosis, a finding that emphasizes the need to measure blood pressure regularly during medical consultations.^19^

The economic evaluation of the hypertension, diabetes and bronchitis treatments indicates that the costs were similar. In this study, expenditure on drugs represented a large proportion of healthcare expenses among patients with diabetes and hypertension, while the expenditure for patients with chronic bronchitis was mostly due to medical consultations. These differences may arise from the possibly lower relative cost of medicine used in the treatment of bronchitis.

The absolute annual cost of antihypertensive drug therapy was lower for diuretics and beta blockers, whether administered as monotherapy or in associations, in comparison with any other drug. Similar low costs relating to diuretics and beta blockers have been described for patients with hypertension from an American primary care center,^8^ rural health centers in Spain,^15^ and participants in a randomized clinical trial.^13^ Monotherapy using calcium channel blockers and ACE inhibitors had the highest cost.^8,12^^,^^20^

The control of hypertension using monotherapy was more frequently attained in patients taking calcium channel blockers (80%) and beta blockers (71%), in comparison with those taking diuretics (54.9%) and ACE inhibitors (52%). Taking into account the cost and effective control of hypertension, the most cost-effective monotherapy was based on diuretics, followed by beta blockers, a finding that is in accordance with the results from other studies,^20,21^ particularly those considering the lifetime duration of treatment.^13^

Clinical trials have demonstrated the efficacy of ACE inhibitors in reducing blood pressure.^22,23^ However, the results of the trials were based on the investigation of selected samples of participants that did not represent the whole population of hypertensive patients. In addition, not all presentations of ACE inhibitors were tested, and it is questionable whether all of them have the same efficacy.^23^

The use of a cost-effectiveness ratio requires the assumptions that the agent tolerability is comparable and that blood pressure lowering is a valid surrogate for cardiovascular risk reduction. In this context, the less advantageous cost-effectiveness ratios for ACE inhibitors and calcium channel blockers, detected in this and in other studies,^12,20^ indicates that they should not be recommended as the first-choice drugs for the treatment of hypertension,^20^ particularly among those without other chronic conditions. Therefore, there are specific groups, such as diabetic patients, for whom this antihypertensive might be the first choice.

The pattern of use for antihypertensive medicines certainly reflects medical prescription behavior^24-26^ as well as inadequate blood pressure control, poor compliance or discontinuation of therapy, and switching between medicines.^21^ Finally, the differences in cost among antihypertensive drug classes become less marked when the costs in relation to quality, adjusted for years of life, are calculated. It should be considered that differences between patients rather than differences between drug prices account for the bulk of the variations.^27^

In conclusion, we identified that the costs of hypertension outside of hospitals are mainly dependent on the expenses with blood pressure-lowering drugs. The treatment of hypertension using diuretics or beta blockers was more cost-effective than the treatment using ACE inhibitors and calcium channel blockers. This finding may allow healthcare planners to make better decisions regarding the allocation of funds^7^ between competing therapeutic options and priorities.^27,28^ This economic evaluation provides a means for making such choices more rational and the allocation of resources more efficient. Nevertheless, despite reasonable concern about the cost of healthcare, it should not take precedence over the quality of and access to care.^29^ The effectiveness of medical assistance is defined as the ability to maintain equity on an efficient basis for the optimization of health and welfare benefits for the population as a whole.^30^
